# Deep Sequencing to Identify the Causes of Viral Encephalitis

**DOI:** 10.1371/journal.pone.0093993

**Published:** 2014-04-03

**Authors:** Benjamin K. Chan, Theodore Wilson, Kael F. Fischer, John D. Kriesel

**Affiliations:** 1 Division of Infectious Diseases, Department of Internal Medicine, University of Utah School of Medicine, Salt Lake City, Utah, United States of America; 2 Department of Pathology, University of Utah School of Medicine, Salt Lake City, Utah, United States of America; Cincinnati Childrens Hospital Medical Center, United States of America

## Abstract

Deep sequencing allows for a rapid, accurate characterization of microbial DNA and RNA sequences in many types of samples. Deep sequencing (also called next generation sequencing or NGS) is being developed to assist with the diagnosis of a wide variety of infectious diseases. In this study, seven frozen brain samples from deceased subjects with recent encephalitis were investigated. RNA from each sample was extracted, randomly reverse transcribed and sequenced. The sequence analysis was performed in a blinded fashion and confirmed with pathogen-specific PCR. This analysis successfully identified measles virus sequences in two brain samples and herpes simplex virus type-1 sequences in three brain samples. No pathogen was identified in the other two brain specimens. These results were concordant with pathogen-specific PCR and partially concordant with prior neuropathological examinations, demonstrating that deep sequencing can accurately identify viral infections in frozen brain tissue.

## Introduction

Current diagnostic methods used in cases of infectious encephalitis successfully identify a specific microbiologic cause of the disease in ∼40% of cases.[1], [2] Recent work suggests that a larger number of cases actually have an infectious etiology but are misdiagnosed.[3]. PCR of CSF can be very helpful for identifying DNA viruses (e.g. herpes simplex virus type 1, HSV1) though it is less effective for the detection of RNA viruses (e.g. West Nile Virus).[4] Further limiting the efficacy of all PCR, culture, and antibody-dependent diagnostic methods are the requirements of specialized reagents and *a priori* knowledge of pathogens to be tested. An incomplete panel of microbial candidates for specific testing can lead to false-negative test results with missed opportunities for effective therapy.[5] Finally, validated PCR primers and protocols sometimes fail to identify known pathogens due to mutations in the primer-binding region, an issue previously addressed by our group in the detection of GB Virus C (GBV-C) in demyelinated human brain.[6]

Deep sequencing offers the prospect of relatively unbiased testing for all previously catalogued and sequenced microbial pathogens in a single test. Where specific PCR, serology and culture focus on a defined set of candidate pathogens, deep sequencing presents a relatively unbiased survey of RNA or DNA sequences present in a sample. Furthermore, this approach does not rely on microbial recovery and isolation, an important attribute given that the microbiome is diverse and, for the most part, cannot be readily cultured.[7] Limitations of the deep sequencing approach for diagnosing infections include: the possible introduction of contaminating sequences into the preparation, difficulties with identifying sequences not included in reference databases (e.g. GenBank) and understanding the significance of rare sequences found within the sample. These problems must be addressed by the use of appropriate controls and, where possible, metagenomic techniques.

**Figure 1:**
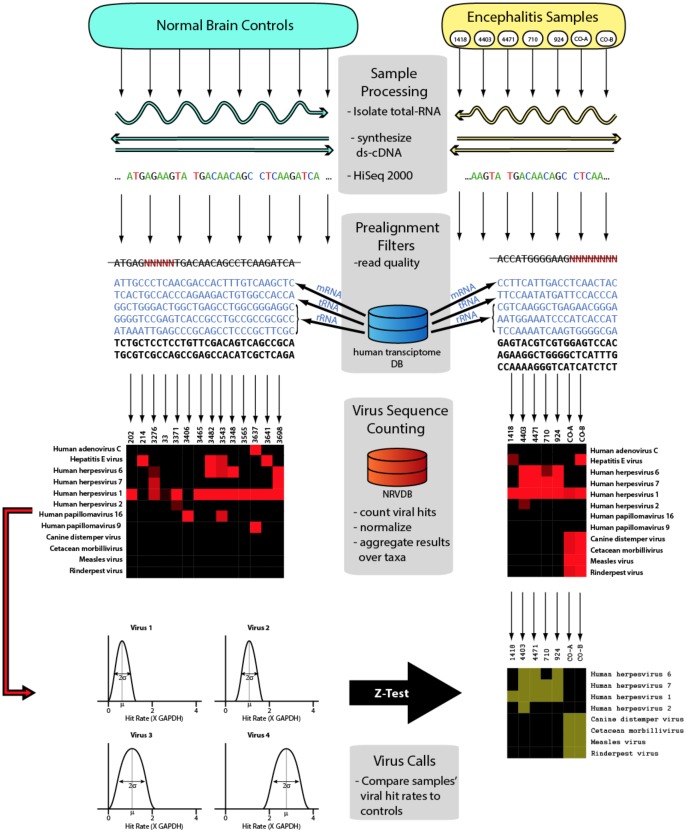
Overview of the deep sequencing analysis pipeline.

In the current study, seven encephalitis cases and fourteen normal brain controls were sequenced and evaluated for the presence of viral sequences. Building upon our recent detection of a novel variant of GBV-C in the brain of an individual who died with primary progressive multiple sclerosis (PPMS), updated bioinformatics methods were used in the current study (Figure 1).[6] Identification of a pathogen was possible in each of the five samples that had a known or strongly suspected infectious etiology, and no pathogen was identified in the two samples without a suspected infectious etiology.

## Methods

### Ethics Statement

This research was submitted to the University of Utah Health Sciences IRB and, since it was performed on de-identified pathologic material, was found to be exempt from review and oversight.

### Samples

Fourteen frozen normal control and 5 frozen encephalitis brain specimens were obtained from the Rocky Mountain and UCLA Brain Banks. Two additional frozen encephalitis specimens were obtained from Dr. Don Gilden at the University of Colorado. All the specimens were collected post-mortem within 20 hours of death, either fresh frozen or snap frozen in liquid nitrogen and were associated with a neuropathological diagnosis. All 7 diseased specimens were from subjects with encephalitis verified by neuropathology. The samples were assigned to one of two groups: controls (n = 14) and encephalitis (n = 7).

### RNA Extraction and RNA-seq

RNA was extracted from frozen brain (volume ∼10 mm^3^) using a Qiagen (Valencia, CA) RNeasy Blood and Tissue kit. RNA was extracted because all viruses utilize RNA at some point during their lifecycle. The extracted RNA was DNase treated per kit instructions and submitted for sequencing at the University of Utah Next Generation Sequencing Shared Resource Facility. Prior to sequencing, RNA was analyzed on an Agilent Bioanalyzer Nanochip (Agilent Technologies, USA) and evaluated for RNA size, abundance and integrity as previously described.[6] Samples were reverse transcribed and prepared with the Illumina TruSeq kit. To ensure the inclusion of possible RNA genomes, oligo dT selection was not performed. To avoid bias, rRNA selection was also not performed. Deep sequencing was performed using two barcoded samples per lane from a single end (50 bases) on an Illumina HiSeq 2000. The sequences have been deposited in the NCBI dbGaP database at URL http://www.ncbi.nlm.nih.gov/projects/gap/cgi-bin/study.cgi?study_id=phs000715.v1.p1. (This link will be activated for controlled access on or about May 1, 2014.)

### Screening of Reads

Metagenomic analysis of the specimens was performed blind, that is without the benefit of pathology reports or other diagnostic information. The sequence data sets were then screened for quality: FASTQ reads containing five or more positions with an Illumina quality score less than 19 were removed and excluded from analysis, providing High Quality (HQ) reads. The number of identical HQ reads for each obtained sequence was noted in a compressed FASTA format to reduce file size and computing run-times in subsequent analysis steps. Using the Bowtie computer program, each HQ read set was aligned to the human genome (NCBI build GRCh37.68) and human transcriptome.[8], [9] Reads that aligned (using Bowtie) to the human genome, human transcriptome, human and mouse ribosomes, or Φ-X174 (an internal sequencing control) were excluded from further analysis. Sequences that did not align to any of those databases were carried forward in the analysis as “screened reads”.

### Non-redundant Viral Database

The sets of screened reads were then aligned to sequences in a non-redundant viral database (NRVDB, http://fischer-lab.path.utah.edu/data/GBV-C/NR_ViroBank) using MegaBLAST.[10] The NRVDB was derived from 1,296,974 viral sequences in the GenBank database. It includes 579,282 unique viral sequence records of 31 to 1.2 million bp in length, representing 2480 different viral taxa. The use of this database reduces redundant hits resulting from overrepresented taxa such as HIV, Hepatitis C Virus and Influenza A Virus, which collectively comprise >50% of the total viral records within GenBank.

### Determination of Hit-Rates and P-Values

Using MegaBLAST with a word size of 28, individual reads that aligned to NRVDB were considered hits. The normalized-hit-rate (NHR) of every sample (encephalitis and controls) to each viral taxon was calculated by dividing the number of sequences that aligned to the taxon by the number of screened reads obtained for the sample. To judge relative enrichment of virus-like sequences, the NHR of each individual encephalitis experimental sample was compared to the NHR distribution for the control samples, for every viral taxon, as previously described.[6] Using custom software written in the Python programming language with SciPy tools, the Z-Test was used to quantify the statistical significance of any viral-taxon overrepresentations in the encephalitis brain samples compared with controls.[11], [12], [13]

### Taxonomy-Based Bioinformatics Follow-Up

Taxa with Bonferroni corrected p-values ≤ 0.01 were analyzed further. MegaBLAST was used to align the screened reads to comprehensive sequence databases of the taxon of interest. Following alignment, contiguous sequences (contigs) were assembled using SSAKE from the reads that aligned to sequences in each taxon of interest.[14] All alignments and contigs were then manually examined to determine whether if they represented human sequence within the taxon-specific database. This determination was based on alignments to the NCBI NR database (MegaBLAST) and examination of the annotations of the GenBank records of the aligning taxon-specific sequences.

### Virus-Specific Amplification

All primer sequences used in this study are given in Document S1. RNA and DNA were re-extracted from the encephalitis and control brain samples (Qiagen, Valencia, CA RNeasy Lipid Tissue and DNeasy Blood and Tissue kits) in preparation for VZV- and HSV-specific PCR and measles-specific RT-PCR. HSV and VZV PCR reactions were performed as previously described.[15], [16] An additional set of HSV1 primers was designed directly from the RNA sequencing data. Reaction conditions were the same as previously described.[16] HSV1 strain 17 syn+ (originally obtained from Dr. James Hill, Louisiana State University) diluted to 10^3^ plaque-forming units/ml was used as the positive HSV control. The VZV postive control material was from a VZV+ MeWo cell culture (kindly provided by Dr. Don Gilden, University of Colorado). The VZV control material was used undiluted. Negative control reactions substituted water for the nucleic acid extracts. Ethidium bromide stained 1.5% agarose gels were used to visualize the resulting PCR products.

Measles virus positive control material was RNA derived from the live-attenuated measles vaccine (MMR-II, Merck & Co, Whitehall Station, New Jersey). Measles virus RNA was extracted from one full dose of the vaccine (0.5 ml, ∼1000 pfu; Qiagen RNeasy, Valencia, CA). A random double stranded cDNA amplicon library was generated using a modified (Document S1) Round A/B protocol.[17] Four μl of extracted measles RNA was used as the round A input and the resulting Round B library was used, undiluted, as the measles positive control. The negative control reaction substituted water for the nucleic acid extracts. The RT-PCR method of Rota et. al. was used to detect measles virus in the experimental and control specimens.[18] Ethidium bromide stained 1.5% agarose gels were used to visualize the resulting RT-PCR products. The PCR and RT-PCR products were purified and Sanger sequenced to confirm the identity of the amplicons (HSV1 or measles).

## Results

### Deep Sequencing

Fifty to 90 million HQ 50 bp reads were obtained from each of the 7 encephalitis samples and 14 normal brain samples, representing RNA present in these brain specimens at the time of collection. Removing reads that aligned to the human genome, transcriptome (NCBI build GRCh37.68), or ribosomes resulted in 199,666 to 907,362 (mean ± SD = 749,443±248,337) screened reads in the encephalitis samples and 216,651 to 2,342,726 (mean ± SD = 944,680±679,117) in the control samples (detailed in Table 1).[19]

**Table 1:** Summary of sequencing results.

	Sample	High Quality Reads	Distinct Sequences	Screened Reads
**Encephalitis**	710	50,126,057	10,149,364	786,777
	924	67,940,913	12390,341	883,231
	1418	76,828,674	15871,022	758,199
	4403	99,541,710	11571,893	833,085
	4471	49,481,868	12206,996	907,362
	CO-A	67,986,609	8996,772	877,782
	CO-B	66,968,038	8,063,517	199,666
**Controls**	33	89,185,458	14,796,756	2,342,726
	202	91,511,792	13,437,573	641,016
	214	94,760,937	12,287,060	752,367
	3276	93,997,180	16,583,547	2,036,850
	3371	96,844,499	15,011,124	2,093,287
	3406	89,328,806	9,378,221	480,061
	3465	105,250,378	6,147,480	556,609
	3482	97,527,486	8,278,798	627,226
	3543	105,242,216	14,172,768	930,838
	3348	102,751,743	4,711,216	528,002
	3565	107,312,994	10,656,003	740,982
	3637	102,581,101	9,910,551	659,917
	3641	85,329,719	8,555,974	618,992
	3698	90,396,566	2,039,260	216,651

High Quality (HQ) Reads includes sequence-identical duplicates. Distinct Sequences does not count duplicate reads. Human transcriptome, human genome, internal sequencing control and human ribosomal sequences are removed from the set of HQ reads to yield Screened Reads.

### Bioinformatic Analysis

For each of the seven encephalitis samples, 2480 taxa were evaluated, providing 17,360 comparisons. A heat map with color intensity representing the negative log transformed and Bonferroni corrected p-values was prepared with Java Treeview (represented in Figure 1).[20] Z-test comparisons of the encephalitis and control brain samples revealed significant viral taxon enrichment in 170 taxon-sample pairs. Each significant pair was systematically evaluated by alignment to both a taxon-specific database and the human genome. A total of 134 sample-taxon pairs, were found to be the consequence of reads that aligned to the human genome, including human endogenous retroviruses, using the lower stringency alignment protocol. These sample-taxon pairs were excluded from further analysis. The remaining 36 taxon-sample pairs were distributed among the 7 encephalitis brain samples (Table 2). These 36 taxon-sample pairs all had p-values <10^−8.5^ after adjusting for multiple comparisons, indicating enrichment. These significantly enriched taxon-sample pairs represented several different viral families; the number of significant pairs is shown in parenthesis: Herpesvirida*e* (17), Paramyxoviridae (10), Poxviridae (6), Hepeviridae (2) and Flaviviridae (1).

**Table 2:** Evaluation of candidate viral taxa.

Brain Sample	Species (taxon)	Human Reads, Annotated as Viral	Non-Human Reads, Presumed Viral	GenBank Sequence Identifier	% GI covered	longest contig (bp)
710	Bovine viral diarrhea virus 3	3	0	390189256	<0.01	0
710	Meleagrid herpesvirus 1	72	0	12025107	0.19	0
710	Human herpesvirus 7	272	0	2746233	1.04	0
710	Human herpesvirus 1	0	10	290766003	0.13	78
1418	Hepatitis E Virus	20	0	319748765	0.87	0
1418	ORF Virus	1	0	325073632	<0.01	0
924	Gallid herpesvirus 3	107	0	10834856	0.26	0
924	Meleagrid herpesvirus 1	72	0	12025107	0.12	0
924	Human herpesvirus 6	354	0	9633069	0.75	0
924	Human herpesvirus 7	296	0	2746233	1.04	0
924	Human herpesvirus 1	0	9	290766003	0.16	78
924	Monkeypox virus	11	0	68449479	0.03	0
4403	Baboon cytomegalovirus	30	0	89994761	0.02	0
4403	Human herpesvirus 5	2	0	37654163	0.02	0
4403	Gallid herpesvirus 3	201	0	10834856	0.26	0
4403	Meleagrid herpesvirus 1	147	0	12025107	0.12	0
4403	Human herpesvirus 6	706	0	9633069	0.74	0
4403	Human herpesvirus 7	562	0	2746233	1.05	0
4403	Human herpesvirus 1	0	35	290766003	0.59	66
4403	Molluscum contagiosum virus	1	0	9628932	<0.01	0
4403	Monkeypox virus	12	0	68449479	0.02	0
4471	Human herpesvirus 6	540	0	9633069	0.74	0
4471	Human herpesvirus 7	454	0	2746233	1.03	0
4471	Monkeypox virus	18	0	68449479	0.02	0
Co-A	Canine distemper virus	2	0	5733642	<0.01	0
Co-A	Cetacean morbillivirus	2	0	38707562	0.24	0
Co-A	Measles virus	0	1067	331784	74.88	215
Co-A	PDPR virus	2	0	71037362	<0.01	0
Co-A	Rinderpest virus	2	0	372001102	0.29	0
Co-B	Hepatitis E virus	17	0	319748765	0.94	0
Co-B	Canine distemper virus	111	0	5733642	0.56	0
Co-B	Cetacean morbillivirus	69	0	38707562	0.37	0
Co-B	Measles virus	0	46434	331784	97.92	4019
Co-B	PDPR virus	0	2	71037362	0.18	0
Co-B	Rinderpest virus	0	131	372001102	0.74	0
Co-B	Monkeypox virus	4	0	68449479	<0.01	0

Viral taxon (species) hit rates from the NRVDB were compared between encephalitis brain samples (N = 7) and control brain samples (N = 14) using the Z-test. Displayed are viral taxa where corrected P<10^−6^ for at least one of the encephalitis samples. Reads aligning to comprehensive taxon-specific databases were examined to determine if they were of viral or human origin (see Methods). Hits to each taxon were assembled into contiguous sequences (contigs) using SSAKE.[14] The GenBank records with the greatest number of alignments were selected, and % GI covered is the amount of the GI covered at ≥ 1X. Taxa where contigs could be formed (i.e. HSV1 and measles) were subjected to further analysis by pathogen-specific amplification.

### Taxon-Specific Follow-Up

Assembly of reads that aligned to the taxon-specific follow up databases resulted in apparently viral contigs ranging from 66 to 4019 bp long in 5 of the 7 encephalitis samples (Table 2). These contigs were re-aligned to the taxon-specific database as well as the human genome with MegaBLAST.

Groups of closely related taxa were identified as significant using this method. For instance, sequence records from several paramyxovirus family members were significantly associated with samples CO-A and CO-B. Considering sequence homologies and human origin of the samples, multiple viral taxa (canine distemper virus, rinderpest virus, cetacean morbillivirus) were excluded from specific PCR follow-up, and the most closely related human virus (also significantly overrepresented) was used for PCR validation. Furthermore, each alignment was examined manually to determine if the aligning GI contained annotated human sequence, or if it was aligned to a human sequence not found in the canonical human genome and human transcriptome. These results and the resulting viral candidates chosen for specific PCR follow-up are shown in Table 2.

### PCR Confirmation of Viral Sequence

**Figure 2:**
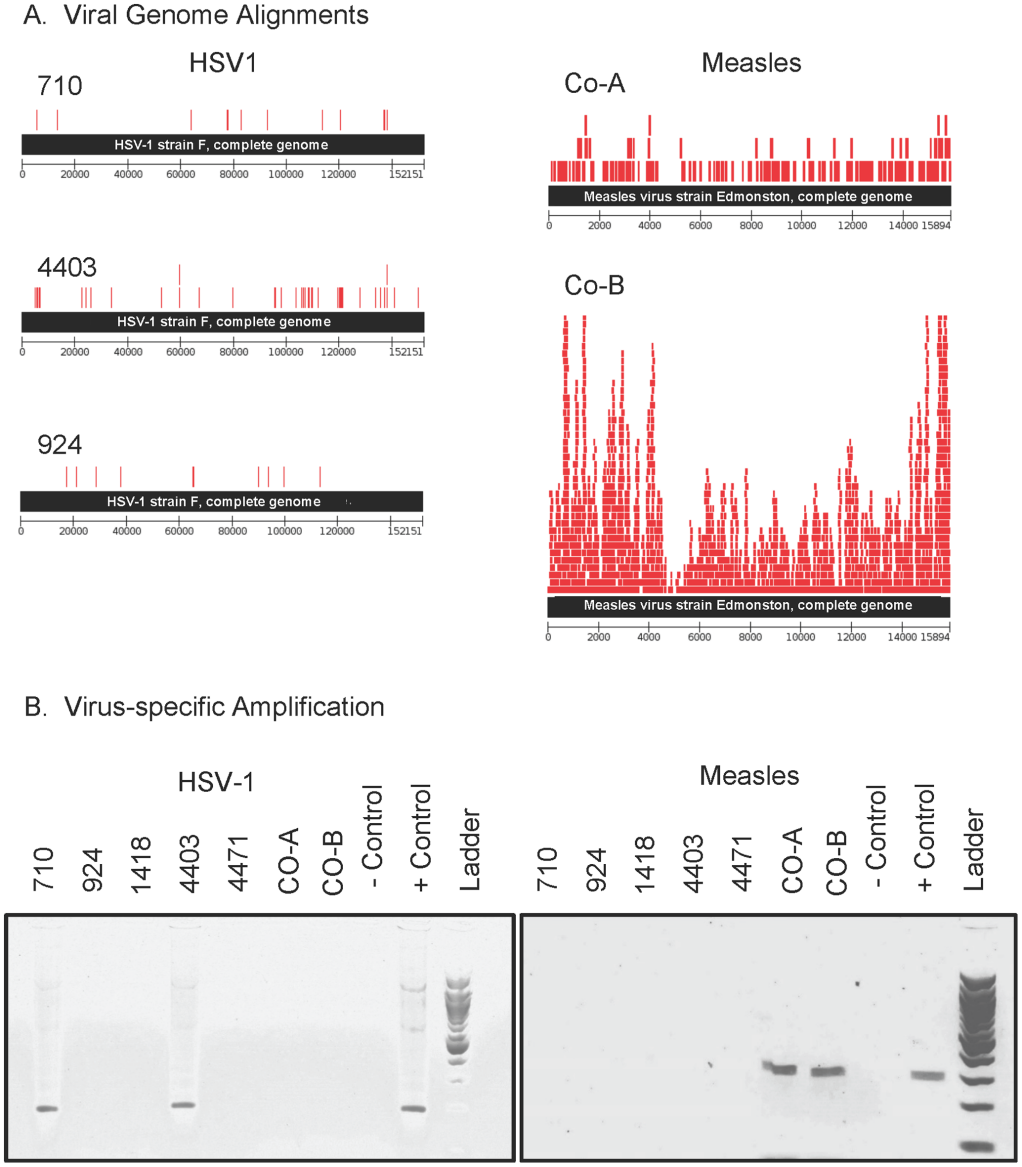
Sequence alignment and experimental confirmation of viral identity. Panel A: 50 bp reads mapping to the HSV1 (strain F, gi: 290766003) genome and measles virus (Edmonston strain, gi: 331784) genome are shown. Panel B: Virus–specific PCR amplicons are shown with the positive control (viral DNA: HSV 17 syn+, clinical strain VZV, MV extracted from vaccine) and negative controls (ddH_2_O). Custom primers were required to amplify HSV1 from specimen 924, confirmed by amplicon sequencing (data not shown).

HSV1 sequences were confirmed by Sanger sequencing of PCR products obtained from brain samples 710 and 4403, both from subjects with herpes encephalitis indicated in neuropathology reports (Figure 2, Table 3). Samples Co-A and Co-B were from subjects with subacute sclerosing panencephalitis (SSPE), according to their associated pathology reports. The deep sequencing analysis indicated the presence of the measles virus (MV). This was confirmed by specific PCR (Figure 2). Furthermore, the depth of sequencing coverage in Co-A and Co-B was sufficient to identify mutations in the genomes of each MV strain known to be present in SSPE-causing isolates. [21] Deep sequencing did not identify any viral candidates in samples 1418 and 4471. Specific PCR and RT-PCR for HSV1, VZV, and MV produced no amplicons in these samples (Table 3).

**Table 3:** Comparison of deep sequencing, molecular and pathology results.

Sample	Collection Year	Neuropathology Diagnoses	Sequencing Call	Molecular Followup			
				HSV1	VZV	MV	Comments
CO-A	Unknown	SSPE	Measles Virus	**-**	**-**	**+**	Confirmed SSPE
CO-B	unknown	SSPE	Measles Virus	**-**	**-**	**+**	Confirmed SSPE
710	1983	HSV encephalitis, leukemia	HSV1	**+**	**-**	**-**	Confirmed HSV Encephalitis
924	1985	VZV encephalitis	HSV1	**+**	**-**	**-**	Misdiagnosed as VZV encephalitis, actually HSV Encephalitis
1418	1988	Chronic encephalitis, unknown etiology	None	**-**	**-**	**-**	No Pathogen Identified
4403	2006	HSV encephalitis, stroke	HSV1	**+**	**-**	**-**	Confirmed HSV Encephalitis
4471	2007	Rasmussen's encephalitis	None	**-**	**-**	**-**	No Pathogen Identified

In four of five cases, virus calls from the bioinformatic analysis of the deep sequencing data and the subsequent pathogen specific amplification were concordant with the prior clinical diagnoses. However, our metagenomic analysis did not reveal the presence of VZV sequence in sample 924, although that sample's neuropathology report, dating from 1985, identified VZV as the likely cause of encephalitis. PCR with validated diagnostic primers also failed to confirm the presence of VZV in the sample (data not shown). The deep sequencing of sample 924 did indicate the presence of HSV1, although the validated diagnostic HSV1 primers used successfully on samples 710 and 4403 [16] failed to yield a PCR product with sample 924. Based on the deep sequencing contig alignments, a novel set of primers was designed. A product of the expected size (∼800 bp, data not shown) was obtained, using methods previously described.[16] The sequence of the product was found to be 100% identical to the major capsid protein gene (UL19) of HSV1 McKrae. Thus, the HSV1 present in the brain of sample 924 may represent a strain with mutations present in region of the DNA polymerase gene amplified by the published diagnostic PCR primers.

DNA extracted from each of the 14 control brain specimens were also interrogated with the set of specific HSV1 and VZV primers. No product was obtained for any of the 14 controls with these primer sets (data not shown). Likewise, RT-PCR interrogation of the 14 control specimens for MV yielded no amplicons.

## Discussion

This study demonstrates the utility of using deep-sequencing to identify viral etiologies in encephalitis. As observed previously, validated PCR primers sometimes fail to amplify agents against which they have been validated.[5], [22] Sample 924 was found to contain HSV1, yet diagnostic HSV primers failed to yield a product. PCR primers derived directly from the metagenomic sequence obtained via deep sequencing were eventually found to be effective. The point of these PCR experiments was simply to validate the deep sequencing results and is not seen as an advance in diagnostic PCR reagents for these viruses *per se*.

In four out of five cases, there was agreement between neuropathology reports and the deep sequencing results. Given the fact that no specific reagents were used in these neuropathological examinations, this may be considered remarkable agreement. However, the lack of specificity inherent in these examinations was illustrated by the discordant result obtained for sample 924, where VZV was suspected and HSV1 was detected by deep sequencing and validated by PCR.

In samples with high titers of virus, the coverage obtained from deep sequencing experiments is sometimes sufficient to elucidate many genomic features of the infectious agent. For example, in samples Co-A and Co-B, the signature genomic mutations associated with defective, SSPE-causing virus were easily observed.

Deep sequencing viral detection methods rely upon alignment of the experimental sequences to known viral sequences. This work used the viral nucleotide sequences in GenBank. Using this approach, identification of a previously unknown virus in a sample is possible only if one or more related viruses are present in the database. The nonredundant viral database used here is comprehensive by current (2013) standards, but will need to be updated as new records are added to GenBank.

Deep sequencing technologies are changing rapidly and the analysis of the deep sequencing data is becoming more efficient. As sequencing technologies improve and become more cost effective, their application in clinical diagnostics may become commonplace, especially for the identification of pathogens.[23], [24], [25] This study demonstrates the feasibility of using deep sequencing to identify viral causes of encephalitis.

## Supporting Information

Document S1 **Specifies the PCR primer sets used in this study.**
(DOCX)
