# Supplementary material for: Deep Sequencing to Identify the Causes of Viral Encephalitis
Source: PLoS One. 2014 Apr 3;9(4):e93993. doi: 10.1371/journal.pone.0093993 (PMC3974838; doi:10.1371/journal.pone.0093993)
Supplement: Document S1 — Specifies the PCR primer sets used in this study. (DOCX) [file pone.0093993.s001.docx]

**Supplementary Document 1 (Chan et al, PLOSONE, 2014)**

**Primer Sets Used in Study**

Measles Nucleoprotein (N) gene

Forward: CATTACATCAGGATCCGG

Reverse: GTATTGGTCCGCCTCATC

Reference:

Rota PA, Khan AS, Durigon E, Yuran T, Villamarzo YS, et al. (1995) Detection of measles virus RNA in urine specimens from vaccine recipients. J Clin Microbiol 33: 2485-2488.

VZV XbaI M Gene

Forward: ATGTCCGTACAACATCAACT

Reverse: CGATTTTCCAAGAGAGACGC

Reference:

Puchhammer-Stockl E, Popow-Kraupp T, Heinz FX, Mandl CW, Kunz C (1991) Detection of varicella-zoster virus DNA by polymerase chain reaction in the cerebrospinal fluid of patients suffering from neurological complications associated with chicken pox or herpes zoster. J Clin Microbiol 29: 1513-1516.

HSV1 DNA Polymerase

Forward: ATCAACTTCGACTGGCCCTT

Reverse: CCGTACATGTCGATGTTCAC

Reference:

Madhavan, H. N., K. Priya, A. R. Anand, and K. L. Therese (1999) Detection of herpes simplex virus (HSV) genome using polymerase chain reaction (PCR) in clinical samples comparison of PCR with standard laboratory methods for the detection of HSV. J. Clin. Virol. 14:145-151.

HSV1 glycoprotein D

Forward: GCCGTCAGCGAGGA

Reverse: TCAGCAAATAGAAG

Reference:

Stevenson J, Hymas W, Hillyard D. (2005) Effect of sequence polymorphisms on performance of two real-time PCR assays for detection of herpes simplex virus. J Clin Microbiol 43: 2391-2398.

HSV1 Major Capsid Protein

Forward: ACGCCCAGGTCCACGTTC

Reverse: GCCAATCTGGTGGCCAA

Reference:

Designed from RNAseq of sample 924

Comments: The PCR product generated from sample 924 was 100% identical to GI: 400530476 nucleotides 36784-37493.

Round-AB

Primer-A_BpuE1: CTTGAGCGTGTGAGCANNNNNNNNN

Primer-B_BpuE1: CTTGAGCGTGTGAGCA
